# ORAI1 inhibition as an efficient preclinical therapy for tubular aggregate myopathy and Stormorken syndrome

**DOI:** 10.1172/jci.insight.174866

**Published:** 2024-03-22

**Authors:** Roberto Silva-Rojas, Laura Pérez-Guàrdia, Alix Simon, Sarah Djeddi, Susan Treves, Agnès Ribes, Lorenzo Silva-Hernández, Céline Tard, Jocelyn Laporte, Johann Böhm

**Affiliations:** 1 Institut de Génétique et de Biologie Moléculaire et Cellulaire (IGBMC), Inserm U1258, CNRS UMR7104, University of Strasbourg, Illkirch, France.; 2 Departments of Neurology and Biomedicine, Basel University Hospital, Basel, Switzerland.; 3 Department of Life Sciences and Biotechnology, University of Ferrara, Ferrara, Italy.; 4 Institute of Metabolic and Cardiovascular Disease, Inserm UMR1297 and University of Toulouse 3, Toulouse, France.; 5 Laboratory of Hematology, University Hospital of Toulouse, Toulouse, France.; 6 Neurology Service, Hospital Universitario Puerta de Hierro Majadahonda, Majadahonda, Madrid, Spain.; 7 University Lille, Inserm, CHU Lille, U1172 Lille Neuroscience & Cognition, Center for Rare Neuromuscular Diseases Nord/Est/Ile-de-France, Lille, France.

**Keywords:** Muscle biology, Therapeutics, Calcium channels, Genetic diseases, Skeletal muscle

## Abstract

Tubular aggregate myopathy (TAM) and Stormorken syndrome (STRMK) are clinically overlapping disorders characterized by childhood-onset muscle weakness and a variable occurrence of multisystemic signs, including short stature, thrombocytopenia, and hyposplenism. TAM/STRMK is caused by gain-of-function mutations in the Ca^2+^ sensor STIM1 or the Ca^2+^ channel ORAI1, both of which regulate Ca^2+^ homeostasis through the ubiquitous store-operated Ca^2+^ entry (SOCE) mechanism. Functional experiments in cells have demonstrated that the TAM/STRMK mutations induce SOCE overactivation, resulting in excessive influx of extracellular Ca^2+^. There is currently no treatment for TAM/STRMK, but SOCE is amenable to manipulation. Here, we crossed *Stim1^R304W/+^* mice harboring the most common TAM/STRMK mutation with *Orai1^R93W/+^* mice carrying an ORAI1 mutation partially obstructing Ca^2+^ influx. Compared with *Stim1^R304W/+^* littermates, *Stim1^R304W/+^Orai1^R93W/+^* offspring showed a normalization of bone architecture, spleen histology, and muscle morphology; an increase of thrombocytes; and improved muscle contraction and relaxation kinetics. Accordingly, comparative RNA-Seq detected more than 1,200 dysregulated genes in *Stim1^R304W/+^* muscle and revealed a major restoration of gene expression in *Stim1^R304W/+^Orai1^R93W/+^* mice. Altogether, we provide physiological, morphological, functional, and molecular data highlighting the therapeutic potential of ORAI1 inhibition to rescue the multisystemic TAM/STRMK signs, and we identified myostatin as a promising biomarker for TAM/STRMK in humans and mice.

## Abstract 2

Inhibition of the Ca^2+^ channel ORAI1 rescues the multi-systemic signs in a mouse model for tubular aggregate myopathy and Stormorken syndrome.

## Graphical abstract

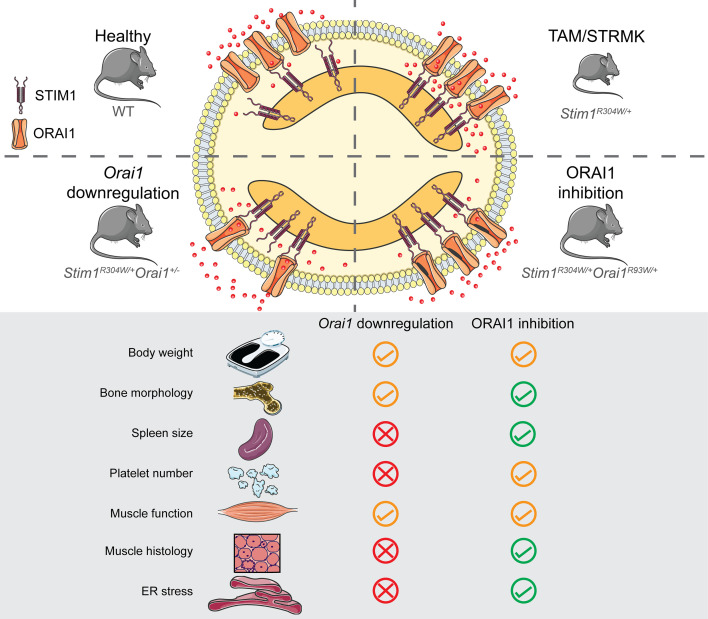

## Introduction

Tubular aggregate myopathy (TAM) is a rare muscle disorder with wide phenotypic heterogeneity, ranging from asymptomatic hyperCKemia to progressive childhood-onset forms with severe upper and lower limb muscle weakness, myalgia, cramps, and contractures (1, 2). Most patients exhibit additional multisystemic signs, such as miosis, thrombocytopenia, hyposplenism, ichthyosis, short stature, and dyslexia, and the full clinical picture is referred to as Stormorken syndrome (STRMK) (2–4).

TAM and STRMK (OMIM #160565 and #615883) form a clinical continuum, and the occurrence and degree of the pathologic signs depend on the implicated gene and the position of the mutation. The majority of patients with TAM/STRMK carry heterozygous missense mutations in *STIM1*, encoding a ubiquitously expressed Ca^2+^ sensor residing at the endoplasmic/sarcoplasmic reticulum (ER/SR). Mutations in the Ca^2+^-sensing EF-hand in the luminal part of STIM1 essentially give rise to a muscle phenotype with occasional depletion of platelets and anomalies of skin, spleen, and bones (5–13), while mutations in the cytosolic coiled-coil domain and more specifically of the arginine 304 residue correlate with a multisystemic presentation of the disorder (8, 10, 14–19). Missense mutations in the plasma membrane Ca^2+^ channel ORAI1 are less common and either affect the pore-forming transmembrane domain and cause a severe phenotype with marked muscle weakness, miosis, bleeding diathesis, ichthyosis, and dyslexia or induce amino acid substitutions in the concentric channel rings and give rise to a more moderate phenotype (18, 20–22). Finally, missense mutations in the muscle-specific SR Ca^2+^-buffering protein calsequestrin (CASQ1) and in the muscle-specific SR Ca^2+^ release channel RyR1 form the mild end of the TAM/STRMK spectrum and were found in patients with adult-onset muscle weakness in the absence of multisystemic signs (23–25).

Ca^2+^ is essentially stored in the ER/SR and serves as a ubiquitous and highly versatile second messenger in all eukaryotes. In response to external stimuli, Ca^2+^ ions are temporarily and spatially released to the cytoplasm where they induce various signaling cascades and exert a plethora of biological processes, including proliferation, neuronal transmission, hormone secretion, or coagulation (26). In myofibers, Ca^2+^ is bound to calsequestrin polymers in the SR and is released through RyR1, and the increase of myoplasmic Ca^2+^ concentrations initiates muscle contraction (27). As a consequence of Ca^2+^ store depletion in both excitable and nonexcitable cells, STIM1 undergoes a conformational change and assembles into oligomers able to activate ORAI1 and trigger extracellular Ca^2+^ entry (28, 29). This fundamental mechanism of Ca^2+^ store refilling, known as store-operated Ca^2+^ entry (SOCE), ensures the maintenance of high intracellular Ca^2+^ gradients for oscillatory Ca^2+^ signaling.

Functional investigations in cellular models have demonstrated that the TAM/STRMK mutations induce overactivation of the SOCE pathway and an excessive extracellular Ca^2+^ influx through gain of function (GoF) (6, 8, 12, 17, 18, 20–23). By contrast, recessive *STIM1* and *ORAI1* loss-of-function (LoF) mutations hamper SOCE, prevent Ca^2+^ store refill, and cause SCID (OMIM # 612782 and #612783), involving chronic infections, autoimmunity, muscular hypotonia, and amelogenesis imperfecta (30–32). To correlate the cellular alterations with disease development, several *Stim1* and *Orai1* mouse models with GoF and LoF mutations have been generated (33–39). SCID mice lacking either STIM1 (*Stim1^–/–^*) or ORAI1 (*Orai1^–/–^*) or carrying a homozygous ORAI1 mutation obstructing Ca^2+^ passage (*Orai1^R93W/R93W^*) are perinatally lethal, while heterozygous *Stim1^+/–^*, *Orai1^+/–^*, and *Orai1^R93W/+^* animals are healthy and fertile (34, 36, 39), demonstrating that the remaining STIM1/ORAI1 level or activity is sufficient to ensure normal SOCE. Among the murine TAM/STRMK models, the *Stim1^R304W/+^* mice, which harbor the most common TAM/STRMK mutation, especially recapitulate the main signs of the human disorder and manifest a lower birth ratio; decreased postnatal growth; structural anomalies of bones, skin, and spleen, thrombocytopenia, and muscle weakness associated with cytosolic Ca^2+^ overload; and enhanced myofiber degeneration (35, 40).

There is currently no treatment for TAM/STRMK, but SOCE is amenable to manipulation, and the availability of a faithful animal model offers the possibility of assessing therapeutic approaches. As the most downstream SOCE component, the Ca^2+^ channel ORAI1 constitutes the principle target for a common therapy for both STIM1- and ORAI1-related TAM/STRMK. To provide the experimental evidence that TAM/STRMK can be prevented by the targeted downregulation of *Orai1*, we previously crossed *Stim1^R304W/+^* mice with *Orai1^+/–^* mice, and the *Stim1^R304W/+^Orai1^+/–^* offspring carrying the recurrent TAM/STRMK mutation and expressing only 50% of ORAI1 underwent extensive phenotyping (41). In contrast to *Stim1^R304W/+^* littermates, *Stim1^R304W/+^Orai1^+/–^* mice were born with the expected Mendelian birth ratio and showed significantly increased postnatal growth and bone architecture and partially improved muscle contraction and relaxation parameters (41). However, the platelet and spleen phenotypes were comparable in *Stim1^R304W/+^Orai1^+/–^* and *Stim1^R304W/+^* mice, exemplifying the incomplete rescue of the multisystemic TAM/STRMK phenotype via ORAI1 downregulation and demonstrating the need for alternative and more efficient therapeutic strategies.

Here, we assessed the therapeutic potential of ORAI1 inhibition by crossing our *Stim1^R304W/+^* model with *Orai1^R93W/+^* mice carrying a heterozygous ORAI1 missense mutation partially obstructing Ca^2+^ influx (37, 39). The *Stim1^R304W/+^Orai1^R93W/+^* offspring were born with a normal Mendelian birth ratio, were larger than the *Stim1^R304W/+^* controls, and displayed relevant improvements of bone structure, muscle function, spleen histology, and platelet count. We also performed comparative RNA-Seq of *Stim1^R304W/+^*, *Stim1^R304W/+^Orai1^+/–^*, and *Stim1^R304W/+^Orai1^R93W/+^* muscle samples and found a major restoration of dysregulated genes in *Stim1^R304W/+^Orai1^R93W/+^* mice especially, concomitant with a significant reduction of resting Ca^2+^ levels. Finally, we identified myostatin as promising biomarker for TAM/STRMK in blood samples from mice and patients.

Overall, this work provides a therapeutic proof of concept and illustrates that the inhibition of ORAI1 activity is a potent method to anticipate the multisystemic signs characterizing TAM/STRMK.

## Results

To date, 4 murine models with different STIM1 GoF mutations in the luminal EF-hands or in the cytosolic coiled-coil domains have been described (33, 35, 38, 42), but only the *Stim1^R304W/+^* mouse harboring the most common TAM/STRMK mutation recapitulates the multisystemic signs of the human disorder (35). Indeed, *Stim1^R304W/+^* mice are smaller than WT littermates, manifest reduced muscle strength, prolonged bleeding times, skin irritations, and spleen anomalies (35) and, thus, represent the most suitable animal model to assess therapeutic approaches.

In a previous attempt to reduce SOCE and extracellular Ca^2+^ entry in our TAM/STRMK mouse model, we crossed *Stim1^R304W/+^* mice with *Orai1^+/–^* mice, but the *Stim1^R304W/+^Orai1^+/–^* offspring showed only a moderate and incomplete rescue of the multisystemic phenotype (41). As an alternative strategy to *Orai1* downregulation, we investigated the therapeutic potential of ORAI1 inhibition. To this aim, we crossed our *Stim1^R304W/+^* model with *Orai1^R93W/+^* mice (39) to obtain *Stim1^R304W/+^Orai1^R93W/+^* offspring carrying an ORAI1 mutation partially impeding Ca^2+^ influx. The WT, *Orai1^R93W/+^*, *Stim1^R304W/+^*, and *Stim1^R304W/+^Orai1^R93W/+^* mice underwent comparative phenotypic characterization, and we examined postnatal growth, bone and spleen morphology, platelet numbers and bleeding time, muscle force and fatigue, resting Ca^2+^ levels and SOCE activity, and skeletal muscle transcriptomics.

### Normalized birth ratio and bone architecture.

The crossing cages containing *Stim1^R304W/+^* and *Orai1^R93W/+^* mice produced a total of 215 WT, *Orai1^R93W/+^*, *Stim1^R304W/+^*, and *Stim1^R304W/+^Orai1^R93W/+^* offspring (Supplemental Figure 1; supplemental material available online with this article; https://doi.org/10.1172/jci.insight.174866DS1). Despite the statistical equiprobability of the 4 genotypes, *Stim1^R304W/+^* pups were significantly underrepresented at only 19.5%, while *Stim1^R304W/+^Orai1^R93W/+^* mice constituted 24.7% of the newborns (*P* = 0.025). This is consistent with our former studies reporting lower birth rates of *Stim1^R304W/+^* mice (35, 41). It also indicates that normal SOCE is critical in embryonic physiology and demonstrates that the reduction of ORAI1 activity overcomes the risk of perinatal lethality of the murine TAM/STRMK model.

**Figure 1:**
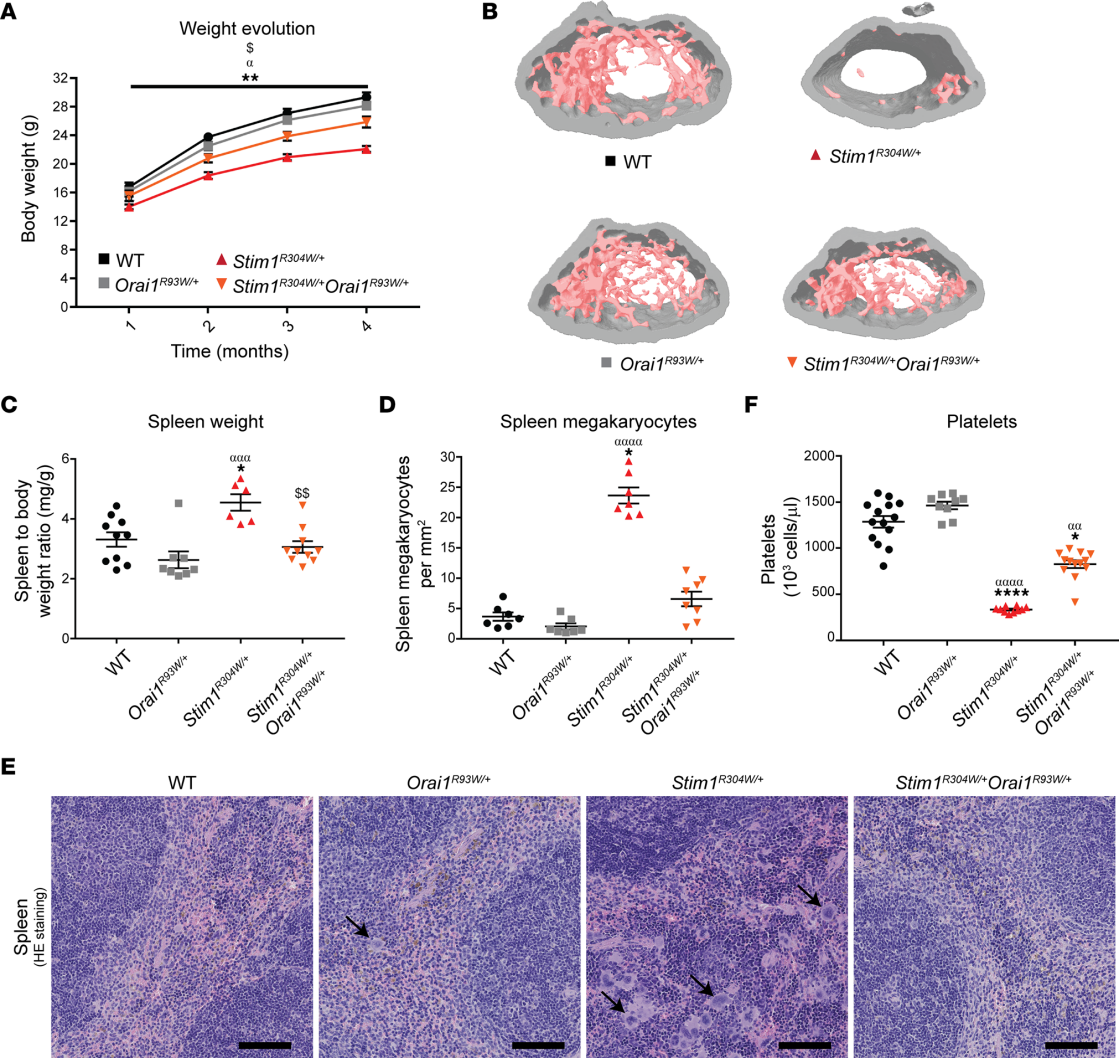
Improved weight gain, bone structure, spleen morphology, and platelet levels in *Stim1^R304W/+^Orai1^R93W/+^* mice. (**A**) Between 1 and 4 months of age, body weight measurements showed a higher growth curve for *Stim1^R304W/+^Orai1^R93W/+^* mice compared with *Stim1^R304W/+^* littermates (*n* = 11–14, 2-way ANOVA and Tukey’s post hoc test). (**B**) 3D reconstruction of the femur microarchitecture illustrated a similar trabecular density in *Stim1^R304W/+^Orai1^R93W/+^* bones and healthy WT and *Orai1^R93W/+^* controls at 4 months (representative images, *n* = 7–8). (**C**–**E**) At 4 months of age, spleen weight, megakaryocyte numbers, and spleen histology (H&E staining) were similar in *Stim1^R304W/+^Orai1^R93W/+^* mice and healthy controls and markedly differed from *Stim1^R304W/+^* littermates (spleen, *n* = 6–10, 1-way ANOVA and Tukey’s post hoc test; megakaryocytes, *n* = 7–8, Kruskal-Wallis and Dunn’s multiple comparison test). Black arrows indicate megakaryocytes. Scale bar: 250 μm. (**F**) Platelet numbers were doubled in *Stim1^R304W/+^Orai1^R93W/+^* mice compared with *Stim1^R304W/+^* littermates at 4 months without reaching WT levels (*n* = 9–14, Kruskal-Wallis and Dunn’s multiple comparison test). Data are shown as the mean ± SEM. Significant differences are indicated as *^,α,$^*P* < 0.05, **^,αα,$$^*P* < 0.01, ^ααα^*P* < 0.001, and ****^,αααα^*P* < 0.0001, with * reflecting comparison of *Stim1^R304W/+^* with the WT group, α comparison with the *Orai1^R93W/+^* group, and ^$^ comparison with the *Stim1^R304W/+^Orai1^R93W/+^* group.

To follow early postnatal development, the offspring underwent regular measurements of body size and weight over the first 4 months. At every time point, *Stim1^R304W/+^* mice were significantly smaller and lighter than WT and *Orai1^R93W/+^* controls, confirming our previous observations (35). In contrast, *Stim1^R304W/+^Orai1^R93W/+^* mice showed continuously higher growth curves, with an increase of body weight and length at 4 months of age compared with *Stim1^R304W/+^* mice (Figure 1A and Supplemental Figure 2A). To investigate the factors contributing to the growth disparities, we examined bone morphology by micro–computerized tomography. By contrast with those from *Stim1^R304W/+^* mice, femurs from *Stim1^R304W/+^Orai1^R93W/+^* mice showed a normal trabecular thickness and bone marrow density, and the overall trabecular structure was comparable with healthy WT and *Orai1^R93W/+^* controls, highlighting a rescue of bone architecture through ORAI1 inhibition (Figure 1B and Supplemental Table 2).

### Normalized spleen histology and increased platelet numbers.

Asplenia or hyposplenism is commonly observed in patients with TAM/STRMK (2, 8, 10, 11, 14–18) and is generally accompanied by the presence of Howell-Jolly-bodies on peripheral blood films. Unlike patients, TAM/STRMK mice manifest splenomegaly associated with megakaryocyte hyperplasia, presumably compromising normal spleen physiology (33, 35, 42). Spleen dissection at 4 months of age confirmed an increased organ weight in *Stim1^R304W/+^* mice compared with healthy controls and revealed a normalization of spleen weight in *Stim1^R304W/+^Orai1^R93W/+^* littermates (Figure 1C). Concomitantly, spleen histology showed a strongly elevated number and abnormal distribution of megakaryocytes in *Stim1^R304W/+^* mice, whereas the spleen morphology and megakaryocyte numbers were indistinguishable between *Stim1^R304W/+^Orai1^R93W/+^* mice and healthy controls (Figure 1, D and E).

Megakaryocytes generate and release platelets into the bloodstream, where they play a pivotal role in thrombus formation in response to vessel wall damage (43). Low platelet numbers in combination with bleeding diathesis is a major clinical feature of TAM/STRMK (2, 7, 8, 10, 11, 14–20, 44), and studies in murine *Stim1* models have shown that platelet preactivation and increased turnover account for the decline of thrombocytes (35, 42). The peripheral blood of *Stim1^R304W/+^* mice contained only 25% of the normal platelet quantity, and the number of circulating platelets was more than doubled in *Stim1^R304W/+^Orai1^R93W/+^* mice (Figure 1F). Accordingly, in vivo assessment of platelet function revealed excessive bleeding times in *Stim1^R304W/+^* mice and a significant improvement in *Stim1^R304W/+^Orai1^R93W/+^* littermates (Supplemental Figure 2B). Although WT values are not reached, these data support a relevant effect of ORAI1 inhibition on the thrombocytopenia phenotype.

### Improved muscle performance and physiology.

Muscle weakness and exercise intolerance constitute the primary clinical signs of TAM/STRMK (3). While the age of onset, disease course, and severity can vary among and between families, first symptoms commonly occur during childhood or adolescence (2). Like many patients, *Stim1^R304W/+^* mice are phenotypically normal at birth and manifest a loss of general and specific muscle force before reaching adulthood (35).

**Figure 2:**
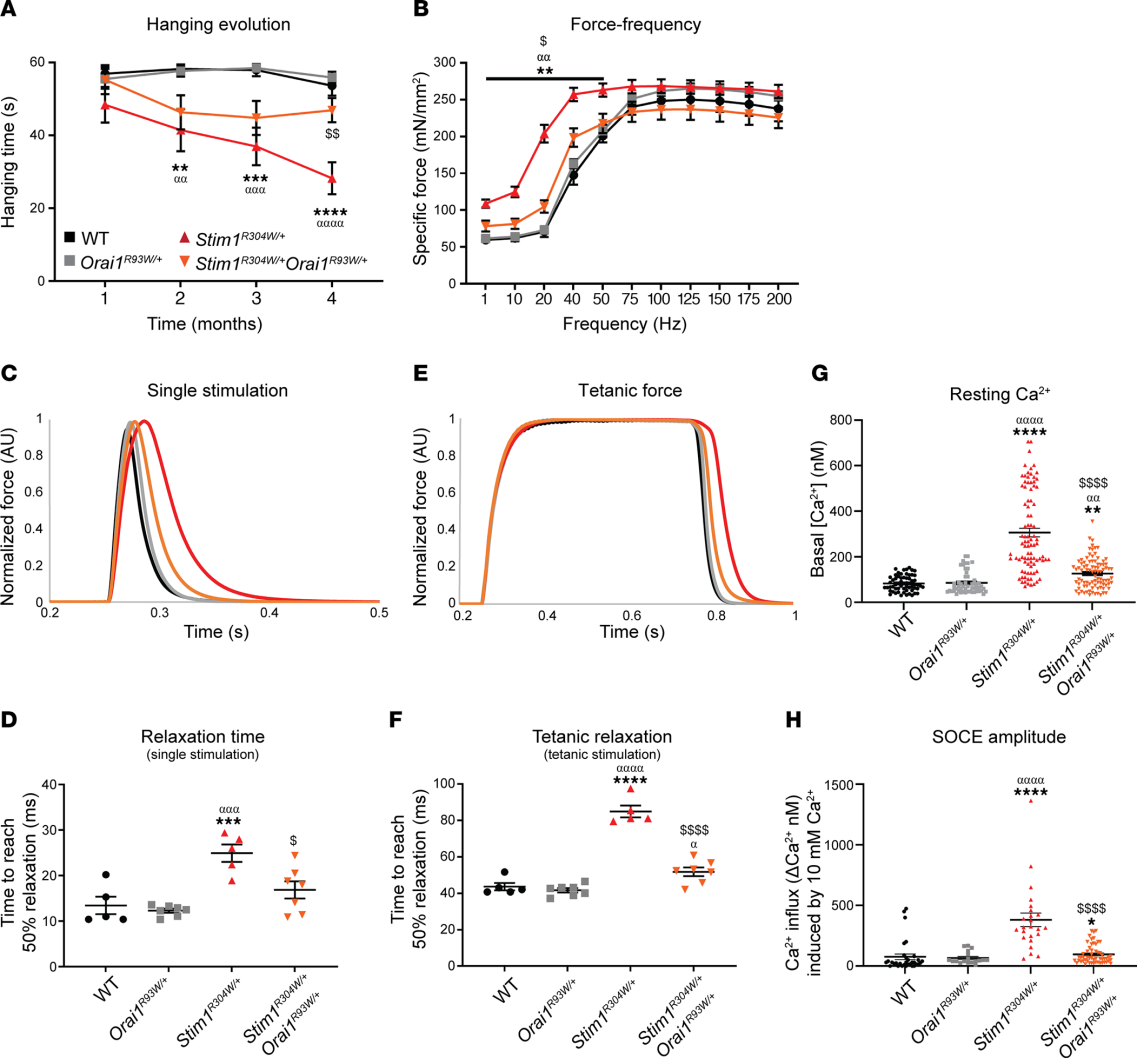
Improved muscle contractility and reduced Ca^2+^ levels in *Stim1^R304W/+^Orai1^R93W/+^* mice. (**A** and **B**) *Stim1^R304W/+^Orai1^R93W/+^* mice outperformed *Stim1^R304W/+^* littermates in the hanging test throughout the first 4 months (*n* = 11–14, 2-way ANOVA and Tukey’s post hoc test). (**B**) Premature muscle contraction of *Stim1^R304W/+^* mice at low stimulation frequencies was normalized in *Stim1^R304W/+^Orai1^R93W/+^* mice at 4 months (*n* = 5–7, 2-way ANOVA and Tukey’s post hoc test). (**C**–**F**) Following single and tetanic stimulations, the relaxation time was significantly delayed in *Stim1^R304W/+^* tibialis anterior and almost normalized in *Stim1^R304W/+^Orai1^R93W/+^* mice at 4 months (mean traces shown, *n* = 5–7, 2-way ANOVA and Tukey’s post hoc test). (**G** and **H**) Resting cytosolic Ca^2+^ levels and SOCE amplitude were strongly increased in *Stim1^R304W/+^* myotubes and shifted toward WT levels in *Stim1^R304W/+^Orai1^R93W/+^* myotubes (resting Ca^2+^, *n* = 53–89 cells; SOCE amplitude, *n* = 18–55 cells; Kruskal-Wallis and Dunn’s multiple comparison test). Data are shown as the mean ± SEM. Significant differences are indicated as *^,α,$^*P* < 0.05, **^,αα,$$^*P* < 0.01, ***^,ααα^*P* < 0.001, and ****^,αααα,$$$$^*P* < 0.0001, with * reflecting comparison of *Stim1^R304W/+^* with the WT group, ^α^ comparison with the *Orai1^R93W/+^* group, and ^$^ comparison with the *Stim1^R304W/+^Orai1^R93W/+^* group.

To assess the effect of ORAI1 inhibition on muscle performance, our mice underwent hanging, open-field, and force transduction tests. Between 1 and 4 months of age, the ability of *Stim1^R304W/+^* mice to hang upside down on a cage grid gradually dropped down to 50% of the normal values, while the performance of *Stim1^R304W/+^Orai1^R93W/+^* littermates remained largely stable with values around 75% of the WT reference (Figure 2A). In line with the augmented general muscle force, *Stim1^R304W/+^Orai1^R93W/+^* mice covered a longer distance with a higher velocity than *Stim1^R304W/+^* littermates in the open-field arena at 10 weeks of age (Supplemental Figure 2, C and D).

We next assessed the in situ muscle contraction and relaxation properties on anesthetized animals through electrical stimulation of the fast-twitch tibialis anterior (TA) muscle. A previous report found higher *Orai1* expression levels in fast-twitch type II than in slow-twitch type I myofibers in mice (45), suggesting that the TA is an appropriate muscle to detect a potential therapeutic effect of ORAI1 inhibition on muscle function. At low stimulation frequencies between 10 and 40 Hz, the force of *Stim1^R304W/+^* muscles increased more rapidly compared with healthy WT and *Orai1^R93W/+^* controls (Figure 2B). In addition to premature muscle contraction, *Stim1^R304W/+^* mice also showed a significant delay in muscle relaxation following single (Figure 2, C and D) and tetanic (Figure 2, E and F) stimulations. Indeed, the relaxation time of *Stim1^R304W/+^* muscle was twice as long as that in the controls and resulted in abnormal fatigue curves (Supplemental Figure 2, E and F). Compared with *Stim1^R304W/+^* littermates, muscle contraction at low stimulation frequencies was normalized in *Stim1^R304W/+^Orai1^R93W/+^* mice (Figure 2B) and muscle relaxation was significantly improved (Figure 2, C–F), demonstrating a positive effect of ORAI1 inhibition on muscle functionality.

To decipher the physiological events leading to normal or pathologic muscle contractility, we isolated primary myoblasts from WT, *Orai1^R93W/+^*, *Stim1^R304W/+^*, and *Stim1^R304W/+^Orai1^R93W/+^* mice and differentiated the mononuclear cells into myotubes. Quantification of the cytosolic Ca^2+^ content and extracellular Ca^2+^ entry revealed a substantial elevation of resting Ca^2+^ and SOCE amplitude in *Stim1^R304W/+^* myotubes compared with WT and *Orai1^R93W/+^* controls (Figure 2, G and H), possibly providing a molecular link with the abnormal muscle contraction and relaxation dynamics in *Stim1^R304W/+^* mice. Accordingly, the decrease of resting Ca^2+^ levels and SOCE amplitude in *Stim1^R304W/+^Orai1^R93W/+^* myotubes compared with *Stim1^R304W/+^* myotubes (Figure 2, G and H) correlated with the normalization of muscle contraction and the improvement of muscle relaxation in *Stim1^R304W/+^Orai1^R93W/+^* mice (Figure 2, B–F).

### Normalized muscle morphology and muscle degeneration markers.

Complementary to the in vivo and in situ muscle force experiments, we dissected the fast-twitch TA and the mixed gastrocnemius muscle of WT, *Orai1^R93W/+^*, *Stim1^R304W/+^*, and *Stim1^R304W/+^Orai1^R93W/+^* mice to examine muscle morphology.

**Figure 3:**
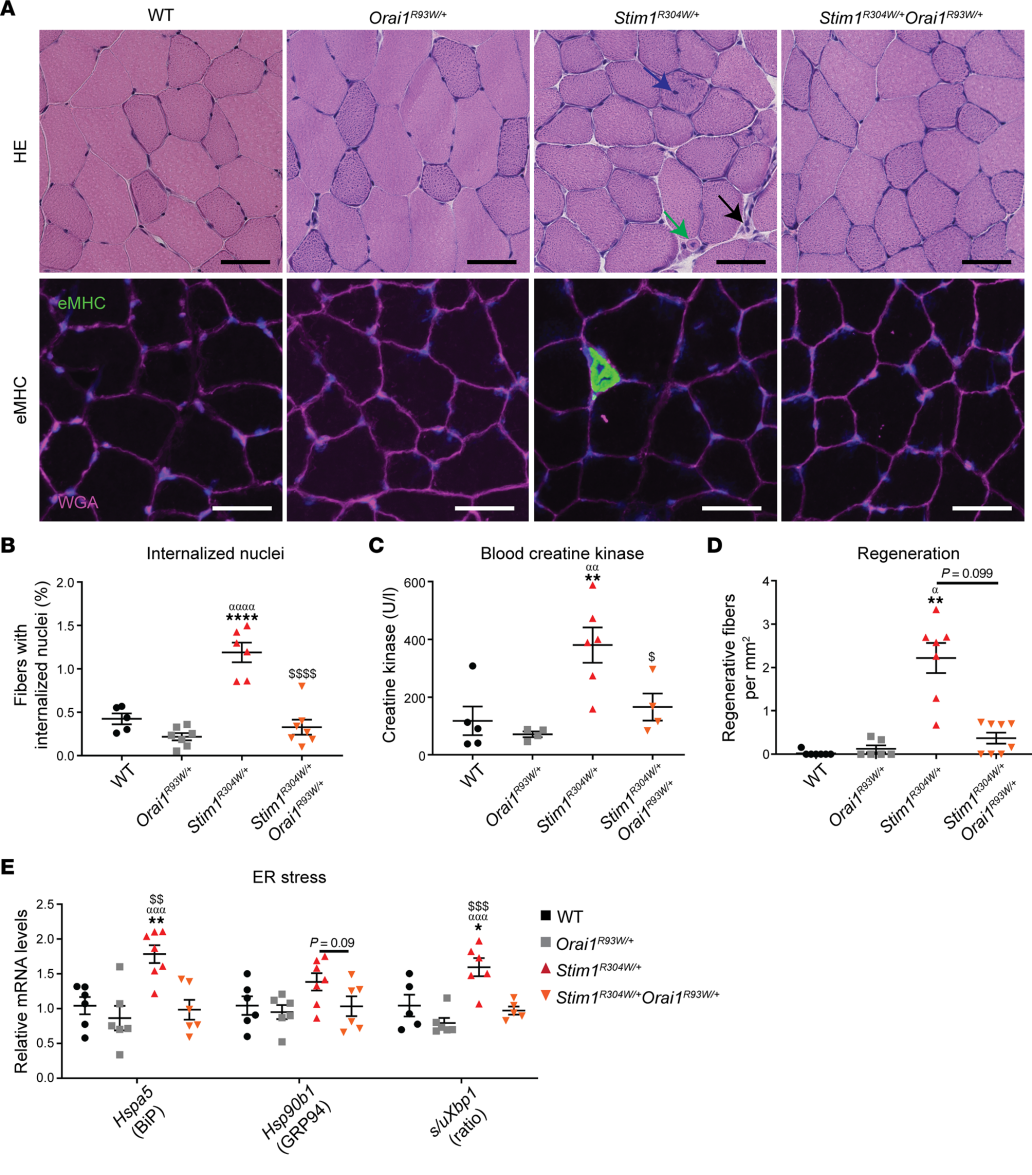
Resolved myofiber degeneration and ER stress in *Stim1^R304W/+^Orai1^R93W/+^* muscle. (**A**) H&E staining of tibialis anterior sections from 4-month-old *Stim1^R304W/+^* mice revealed internalized nuclei (blue arrow), regenerating fibers (green arrow), and immune cell infiltrations (black arrow), while the *Stim1^R304W/+^Orai1^R93W/+^* histology was indistinguishable from that of the WT and *Orai1^R93W/+^* controls (representative images, *n* = 5–7). Immunofluorescence detected prominent embryonic myosin (eMHC) signals, indicating regenerating fibers in *Stim1^R304W/+^* muscle sections and, to a much lesser extent, in WT, *Orai1^R93W/+^*, and *Stim1^R304W/+^Orai1^R93W/+^* myofibers. Scale bar: 50 μm (representative images, *n* = 5–7). (**B**) Quantification of myofibers with internal nuclei showed an increased ratio in *Stim1^R304W/+^* tibialis anterior compared with WT, *Orai1^R93W/+^*, and *Stim1^R304W/+^Orai1^R93W/+^* muscle sections (*n* = 5–8, 1-way ANOVA and Tukey’s post hoc test). (**C**) Serum creatine kinase levels were significantly reduced in 4-month-old *Stim1^R304W/+^Orai1^R93W/+^* blood compared with *Stim1^R304W/+^* samples (*n* = 4–6, 1-way ANOVA and Tukey’s post hoc test). (**D**) Quantification of eMHC signals disclosed an enhanced proportion of regenerating myofibers in *Stim1^R304W/+^* mice and a complete normalization in *Stim1^R304W/+^Orai1^R93W/+^* mice (*n* = 6–8, Kruskal-Wallis and Dunn’s multiple comparison test). (**E**) The expression of the UPR markers *Hspa5* and *Hsp90b1* and the ratio of spliced/unspliced *Xbp1* were comparable in muscle extracts from *Stim1^R304W/+^Orai1^R93W/+^* mice and healthy WT and *Orai1^R93W/+^* controls (*n* = 5–7, 1-way ANOVA and Tukey’s post hoc test). Data are shown as the mean ± SEM. Significant differences are indicated as *^,α,$^*P* < 0.05, **^,αα,$$^*P* < 0.01, ^ααα,$$$^*P* < 0.001, and ****^,αααα,$$$$^*P* < 0.0001, with * reflecting comparison of *Stim1^R304W/+^* with the WT group, α comparison with the *Orai1^R93W/+^* group, and $ the comparison with *Stim1^R304W/+^Orai1^R93W/+^* group.

Histological analyses of *Stim1^R304W/+^* TA and gastrocnemius samples showed myofiber atrophy, abnormal nuclear internalization, and the infiltration of immune cells, all hallmarks of myofiber degeneration (Figure 3, A and B, and Supplemental Figure 3, A and B). In contrast, muscle sections from *Stim1^R304W/+^Orai1^R93W/+^* mice did not show any difference compared with WT mice and no signs of inflammation and myofiber degeneration, highlighting the rescue of skeletal muscle integrity.

In *Stim1^R304W/+^* mice, myofiber degeneration is accompanied by elevated serum creatine kinase (CK) levels and enhanced regeneration, as illustrated by an increased number of fibers expressing embryonic myosin heavy chain (eMHC) (35). In contrast, CK levels in most *Stim1^R304W/+^Orai1^R93W/+^* blood samples were within normal ranges (100–200 U/L) (Figure 3C), and immunofluorescence experiments on *Stim1^R304W/+^Orai1^R93W/+^* muscle sections showed a normal ratio of eMHC-positive myofibers (Figure 3, A and D), confirming the absence of enhanced myofiber degeneration and regeneration cycles. This is also supported by the analysis of unfolded protein response (UPR) markers. We previously showed that the increased cytosolic Ca^2+^ levels in *Stim1^R304W/+^* muscle induce reticular Ca^2+^ stress and trigger UPR, which ultimately leads to myofiber degeneration (40). Quantification of selected UPR markers in TA muscle extracts confirmed an increased expression of the chaperone genes *Hspa5* and *Hsp90b1* and an increased ratio of spliced versus unspliced *Xbp1* in *Stim1^R304W/+^* mice and showed a normalization of the expression levels and splicing ratio in *Stim1^R304W/+^Orai1^R93W/+^* littermates (Figure 3E), demonstrating that Ca^2+^ stress was fully resolved.

### ORAI1 inhibition is more efficient than Orai1 downregulation.

As the first therapeutic strategy to treat TAM/STRMK, we previously investigated the potential of *Orai1* downregulation, and the systematic characterization of *Stim1^R304W/+^Orai1^+/–^* mice revealed an amelioration of several but not all phenotypes affecting bones, skeletal muscle, spleen, and platelets (41). Here, we tested a different approach, and the genetic inhibition of the Ca^2+^ channel ORAI1 substantially improved or rescued all signs of the multisystemic TAM/STRMK phenotype in the *Stim1^R304W/+^* mouse model.

**Figure 4:**
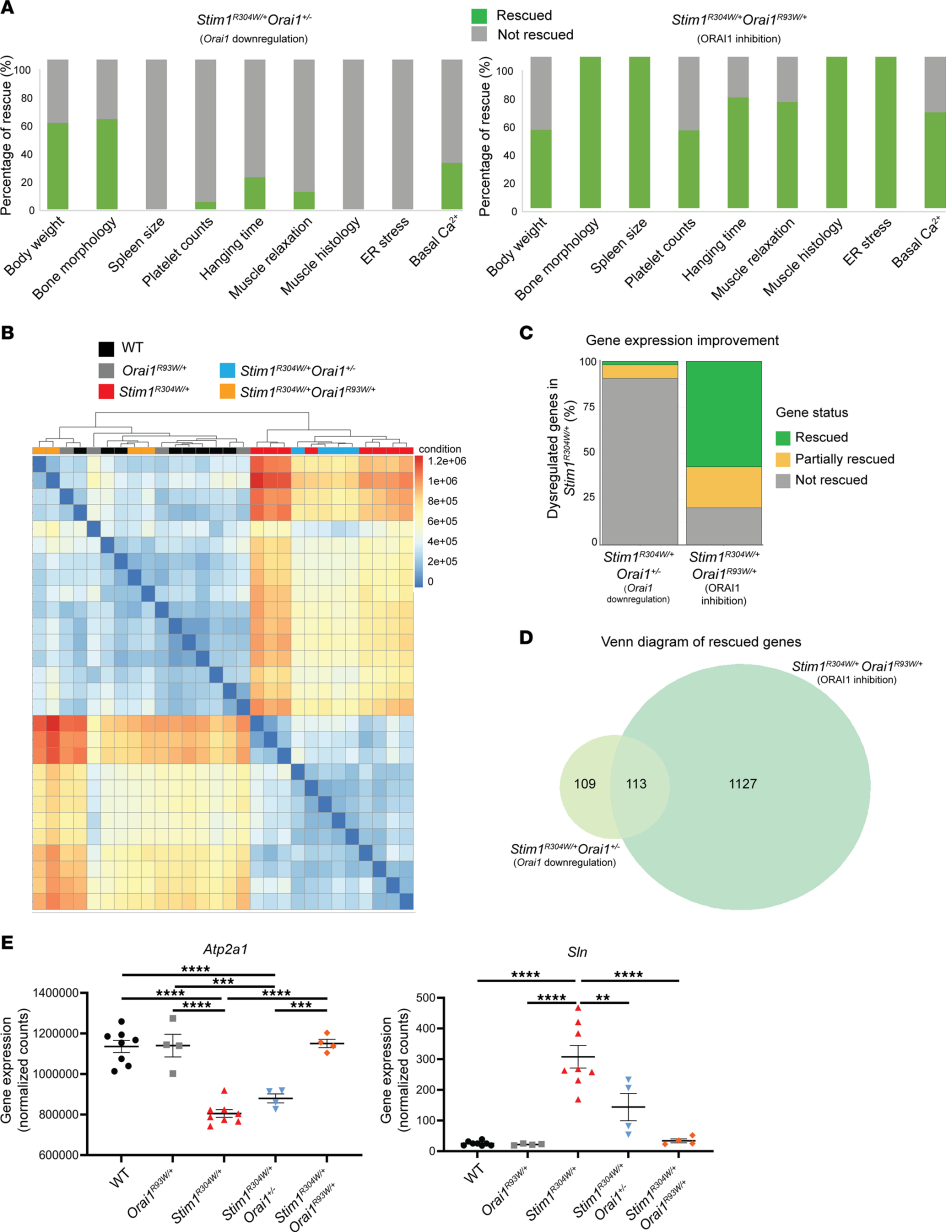
Comparison of ORAI1 inhibition and *Orai1* downregulation on the TAM/STRMK phenotype and transcriptome. (**A**) ORAI1 inhibition (*Stim1^R304W/+^Orai1^R93W/+^* mice) provided a higher overall rescue level of bone and spleen morphology, platelet numbers, muscle histology, contractility, and cytosolic Ca^2+^ content in TAM/STRMK mice compared with *Orai1* downregulation (*Stim1^R304W/+^Orai1^+/–^* mice). The WT phenotype was set at 100% and the *Stim1^R304W/+^* phenotype at 0%. (**B**) Hierarchical clustering of tibialis anterior RNA-Seq data revealed sample grouping of WT, *Orai1^R93W/+^*, and *Stim1^R304W/+^Orai1^R93W/+^* mice on one side and of *Stim1^R304W/+^* and *Stim1^R304W/+^Orai1^+/–^* mice on the other side, confirming the higher therapeutic potential of ORAI1 inhibition at the transcriptomic level (*n* = 4). (**C**) The percentage of genes with improved or rescued expression was substantially higher in *Stim1^R304W/+^Orai1^R93W/+^* mice (ORAI1 inhibition) compared with that in *Stim1^R304W/+^Orai1^+/–^* mice (*Orai1* downregulation). (**D**) Venn diagram illustrating that *Stim1^R304W/+^Orai1^+/–^* (*Orai1* downregulation) and *Stim1^R304W/+^Orai1^R93W/+^* (ORAI1 inhibition) muscle samples shared 113 genes with partially or completely normalized expression. (**E**) Normalized expression of *Atp2a1* and *Sln* in *Stim1^R304W/+^Orai1^R93W/+^* compared with *Stim1^R304W^* and *Stim1^R304W/+^Orai1^+/–^* muscle samples (*n* = 4–9, 1-way ANOVA and Tukey’s post hoc test). Data are shown as the mean ± SEM. Significant differences are indicated as **P < 0.01, ***P < 0.001, and ****P < 0.0001.

To provide an overview of the therapeutic effect of ORAI1 inhibition versus *Orai1* downregulation, we compared body weight, bone morphology, spleen size, platelet quantity, hanging time, muscle relaxation, muscle histology, and ER stress of *Stim1^R304W/+^Orai1^R93W/+^* and *Stim1^R304W/+^Orai1^+/–^* mice in a single diagram, with the *Stim1^R304W/+^* references set at 0% and the WT references at 100% (Figure 4A). All phenotypic and physiological TAM/STRMK parameters were improved through ORAI1 inhibition, and all except body weight attained higher rescue levels compared with *Orai1* downregulation, pointing out that prospective therapies should target ORAI1 activity over *Orai1* expression.

To correlate skeletal muscle function, morphology, and physiology with Ca^2+^ homeostasis, we assessed resting Ca^2+^ levels and SOCE in differentiated myotubes from *Stim1^R304W/+^Orai1^R93W/+^* and *Stim1^R304W/+^Orai1^+/–^* mice (Supplemental Figure 4). In line with the incomplete phenotypic rescue of the animals, *Stim1^R304W/+^Orai1^+/–^* myotubes displayed only a moderate decrease of cytosolic Ca^2+^ levels and extracellular Ca^2+^ entry compared with *Stim1^R304W/+^* mice. In contrast, the resting Ca^2+^ levels were close to normal in *Stim1^R304W/+^Orai1^R93W/+^* myotubes, and the SOCE amplitude was indistinguishable from the WT myotubes.

To determine the benefit of ORAI1 inhibition and *Orai1* downregulation on TAM/STRMK at the molecular level, we performed RNA-Seq on WT, *Stim1^R304W/+^*, *Orai1^R93W/+^*, *Stim1^R304W/+^Orai1^R93W/+^*, and *Stim1^R304W/+^Orai1^+/–^* TA muscle samples (Figure 4B). Hierarchical clustering of the sequencing data revealed a separate sample grouping of WT and *Stim1^R304W/+^* samples, with an upregulation or downregulation of more than 1,200 genes in the TAM/STRMK mouse model compared with the WT. The *Stim1^R304W/+^* and *Stim1^R304W/+^Orai1^+/–^* samples largely clustered together and revealed a rescue of only 1.8% of the dysregulated genes, while the expression of 7.5% of the genes at least partially shifted toward the WT values (Figure 4C). In contrast, *Stim1^R304W/+^Orai1^R93W/+^* mice widely clustered with the healthy WT and *Orai1^R93W/+^* controls. The full rescue of 57.3% of the genes and the partial normalization of 22.2% of the genes attest the significantly higher therapeutic effect of ORAI1 inhibition compared with *Orai1* downregulation.

To retrieve the functional profile of the normalized genes and to define the pathways implicated in the phenotypic rescue of the TAM/STRMK mice, we performed gene ontology term enrichment analyses. The *Stim1^R304W/+^Orai1^R93W/+^* and *Stim1^R304W/+^Orai1^+/–^* mice shared 113 rescued genes with an enrichment of gene ontology terms associated with muscle differentiation and contraction (Figure 4D and Supplemental Figure 5). As the main difference between both therapy cohorts, only the *Stim1^R304W/+^Orai1^R93W/+^* mice displayed a rescue of genes implicated in ER/SR stress and immune response (Supplemental Figure 5B). This is of particular interest since myofiber degeneration involves immune cells to mediate muscle fiber clearance (46) and emphasizes the protective effect of ORAI1 inhibition against TAM/STRMK-typical myofiber degeneration and regeneration (40). Of note, the *Stim1^R304W/+^Orai1^R93W/+^* muscle samples featured a normalized expression of *Atp2a1* and *Sln*, encoding the Ca^2+^ pump SERCA and the negative SERCA regulator sarcolipin, respectively (Figure 4E). These data indicate that Ca^2+^ may be more efficiently removed from the cytosol, which presumably contributes to the improved muscle relaxation of *Stim1^R304W/+^Orai1^R93W/+^* myofibers (Figure 2, C–F).

### Myostatin as circulating biomarker in TAM/STRMK mice and patients.

**Figure 5:**
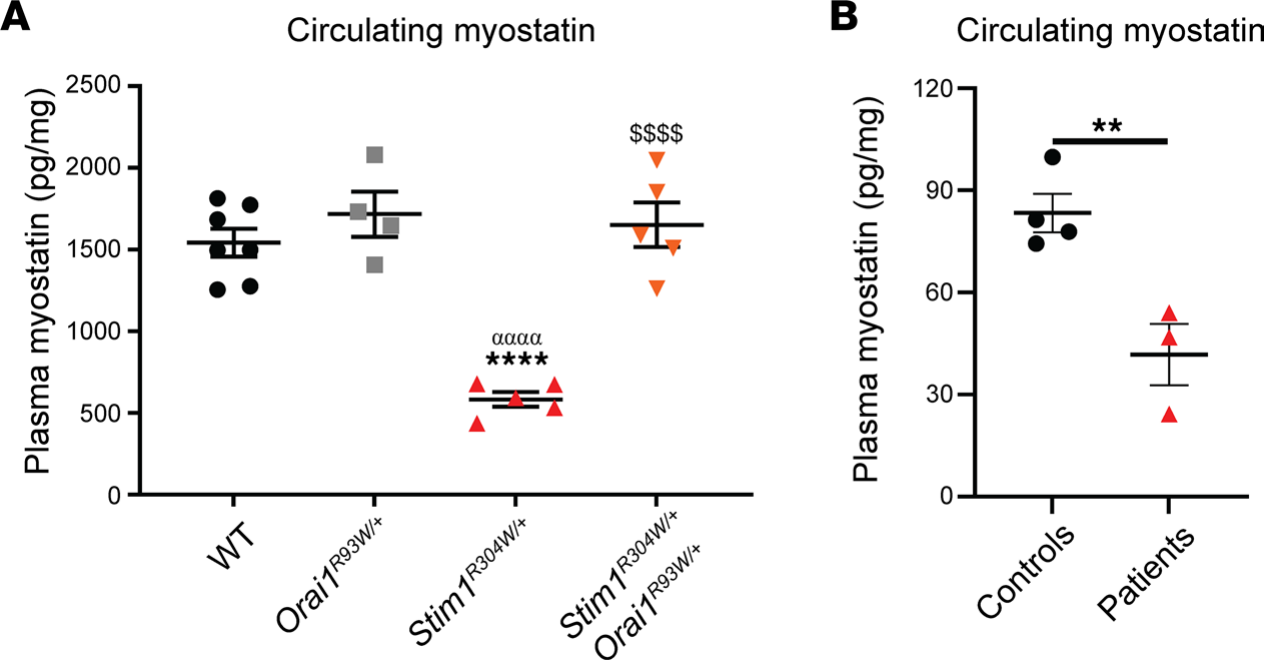
Myostatin is a promising biomarker for TAM/STRMK in mice and patients. (**A**) Circulating myostatin levels were significantly lower in 4-month-old *Stim1^R304W/+^*mice compared with healthy WT and *Orai1^R93/W+^* controls, and they were fully rescued in *Stim1^R304W/+^Orai1^R93/W+^* mice (*n* = 4–7, 1-way ANOVA and Tukey’s post hoc test). (**B**) Analogously to mice, myostatin levels were decreased in patients with TAM/STRMK harboring different *STIM1* mutations compared with healthy controls (*n* = 3–4, *t* test). Data are shown as the mean ± SEM. Significant differences are indicated as **P < 0.01 and ****^,αααα,$$$$^*P* < 0.0001, with * reflecting comparison of *Stim1^R304W/+^*/TAM/STRMK mice and patients with the WT/control group, ^α^ the comparison with the *Orai1^R93W/+^* group, and ^$^ the comparison with the *Stim1^R304W/+^Orai1^R93W/+^* group.

Circulating biomarkers are of major medical importance for following disease progression and evaluating therapeutic efficiencies in noninvasive or minimally invasive ways, and they take a central role in clinical trials for neuromuscular disorders (47). It has previously been shown that CK levels are increased 10-fold in patients with TAM/STRMK and mice and correlate with myofiber degeneration (2, 33, 35), and here, we describe a significant reduction of the serum CK levels in *Stim1^R304W/+^Orai1^R93W/+^* mice (Figure 3C). To complement the CK measurements and to provide an additional circulating biomarker for TAM/STRMK, we compared the list of dysregulated and rescued genes in our RNA-Seq data from WT, *Stim1^R304W/+^*, and *Stim1^R304W/+^Orai1^R93W/+^* mice, and we focused on genes expressed in skeletal muscle and coding for proteins secreted into the bloodstream and detectable in the sera of 4-month-old WT mice (48, 49). *Actc1*, *Alad*, *Anxa2*, *Clic4*, *Gpx1*, *Mstn*, *Sod3*, and *Thbs1* were all differentially expressed in *Stim1^R304W/+^* muscles compared with the WT and *Orai1^R93W/+^* controls, and the vast majority was normalized in *Stim1^R304W/+^Orai1^R93W/+^* mice (Supplemental Figure 6). Owing to its role as major regulator of muscle growth and its utility as biomarker in diverse myopathies (50–52), we selected myostatin (*Mstn*). Using an ELISA test, the level of circulating myostatin was significantly decreased in *Stim1^R304W/+^* plasma compared with that of healthy controls and normalized in *Stim1^R304W/+^Orai1^R93W/+^* samples (Figure 5A). To assess the translational potential of our findings, we next applied the ELISA test to patients with TAM/STRMK carrying different STIM1 mutations (H72Q, I115F, R304W) (Figure 5B). In all 3 patients, the myostatin levels were significantly decreased, suggesting that myostatin may serve as a suitable biomarker for TAM/STRMK in both humans and mice.

## Discussion

TAM and STRMK are spectra of the same multisystemic disorder affecting skeletal muscle, bones, spleen, and platelets. No therapy is available for TAM/STRMK to date, and the absence of a treatment represents an important burden for the affected families and an unmet medical need. TAM/STRMK is caused by excessive extracellular Ca^2+^ influx, and the genetic downregulation of the plasma membrane Ca^2+^ channel ORAI1 in *Stim1^R304W/+^Orai1^+/–^* mice only partially improved the multisystemic disease signs (41), pointing out the necessity for alternative therapeutic strategies. Here, we provided functional, physiological, structural, biochemical, and molecular data demonstrating that the inhibition of ORAI1 significantly reduced Ca^2+^ influx and increased postnatal weight gain, muscle force, and platelet numbers in *Stim1^R304W/+^Orai1^R93W/+^* mice, and fully rescued birth ratio, bone architecture, spleen histology, as well as myofiber morphology and turnover.

### ORAI1 inhibition versus Orai1 downregulation — a measurable difference in efficacy.

The Ca^2+^ channel ORAI1 operates downstream of the Ca^2+^ sensor STIM1 and, thus, constitutes the prime target for therapeutic approaches for the main TAM/STRMK forms — either through the regulation of its expression or through the regulation of its activity.

We assessed the therapeutic potential of both strategies in our murine *Stim1^R304W/+^* model and systematically examined the phenotype of *Stim1^R304W/+^Orai1^+/–^* mice expressing 50% ORAI1 (41) and of *Stim1^R304W/+^Orai1^R93W/+^* mice expressing an ORAI1 mutant with constricted pore (this study). Both *Stim1^R304W/+^Orai1^+/–^* and *Stim1^R304W/+^Orai1^R93W/+^* mice showed a normalized birth ratio, an improvement of trabecular bone structure associated with higher growth curves compared with *Stim1^R304W/+^* littermates, and an amelioration of muscle function and structure. However, the muscle contraction and relaxation dynamics were only moderately amended in *Stim1^R304W/+^Orai1^+/–^* mice (41) and almost rectified in *Stim1^R304W/+^Orai1^R93W/+^* mice, indicating a superior therapeutic efficiency of ORAI1 inhibition over *Orai1* downregulation. This is furthermore supported by the higher general muscle force of *Stim1^R304W/+^Orai1^R93W/+^* mice, the absence of enhanced SR stress and myofiber degeneration, the important reduction of cytosolic Ca^2+^ levels, and the transcriptional normalization of numerous dysregulated genes in skeletal muscle. Moreover, none of the *Stim1^R304W/+^Orai1^R93W/+^* mice exhibited splenomegaly or an abnormal spleen histology, and all displayed a marked increase of thrombocytes compared with *Stim1^R304W/+^* and *Stim1^R304W/+^Orai1^+/–^* mice. Taken together, our findings illustrate that ORAI1 inhibition resolves the multisystemic TAM/STRMK phenotype to a substantially higher degree compared with *Orai1* downregulation and point to pharmacological treatment options targeting ORAI1 conduction and lowering Ca^2+^ influx for prospective clinical trials.

In this context, the biphenyl-triazole CIC-39 has recently been described as a SOCE inhibitor and efficiently reduced extracellular Ca^2+^ entry in fibroblasts derived from patients with TAM/STRMK (53). The CIC-39 treatment of *Stim1^I115F/+^* mice, exhibiting an incomplete TAM/STRMK phenotype, restored the quantity of circulating platelets and minimized bleeding diathesis (54), sustaining the idea that a steric hindrance of SOCE and ORAI1 through pharmacological compounds likely represent the most promising way to treat TAM/STRMK.

### ORAI1 inhibition versus Orai1 downregulation — a different physiological effect.

The ORAI1 Ca^2+^ channel works as a hexamer, and each ORAI1 subunit is composed of 4 α-helical transmembrane domains, with M1 constituting the channel pore and M2–M4 shaping concentric rings surrounding the pore (55–57). In *Stim1^R304W/+^Orai1^+/–^* mice, the 50% reduction of available ORAI1 monomers to form functional Ca^2+^ channels mitigated the pathogenic impact of SOCE overactivity in the affected tissues, but only partially improved the skeletal muscle phenotype and turned out to be ineffective in spleen and platelets (41). It is possible that most or all remaining ORAI1 hexamers in *Stim1^R304W/+^Orai1^+/–^* mice are exposed to constitutive activation through the STIM1 R304W mutant, which may counteract the therapeutic effect of *Orai1* downregulation and explain the incomplete rescue. The disparate improvement levels of the affected *Stim1^R304W/+^Orai1^+/–^* tissues possibly reflect a different Ca^2+^ sensitivity and suggest that, especially, spleen cells and the spleen-derived thrombocytes may require a more stringent control of Ca^2+^ balance compared with bone or skeletal muscle.

In contrast to *Stim1^R304W/+^Orai1^+/–^* mice, the totality of the TAM/STRMK phenotypes was strongly improved or fully resolved in *Stim1^R304W/+^Orai1^R93W/+^* mice. The R93 residue (corresponding to R91 in humans) is located at the narrowest part of the pore, and the substitution of arginine by the large amino acid tryptophan sterically restrains ion passage (30). Depending on the relative proportion of ORAI1 WT and R93W monomers forming the hexamers in heterozygous *Stim1^R304W/+^Orai1^R93W/+^* mice, the Ca^2+^ channels may be obstructed to a variable extent, and the overall reduction of Ca^2+^ entry manifestly sufficed to enable normal or nearly normal physiological processes in skeletal muscle, bones, spleen, and platelets. Moreover, the human ORAI1 R91W mutation was also shown to impede the interaction with STIM1 in a cellular model (58), which depicts another mechanism attenuating SOCE and positively distinguishes ORAI1 inhibition from *Orai1* downregulation.

### Targeting ORAI1 in other Ca^2+^-related diseases.

SOCE overactivation resulting in excessive extracellular Ca^2+^ entry is also reported in other muscle disorders. As an example, the dystrophin-deficient mdx mouse model of Duchenne muscular dystrophy shows high *Orai1* expression levels associated with increased SOCE activity (59). And malignant hyperthermia (MH), characterized by a life-threatening sensitivity to halogenated anesthetics and caused by Ca^2+^ leakage from the SR (60), implies sustained SOCE activation presumably amplifying disease severity (61). Remarkably, the exogenous expression of a dominant-negative ORAI1 mutant reduced the dystrophic features in mdx muscle samples (62, 63) and the cytoplasmic Ca^2+^ concentrations in MH mice-derived muscle cells (64). Similarly, treatment of murine MH cells with the unspecific SOCE inhibitors BTP-2, Gd^3+^, or GsMTx-4 decreased Ca^2+^ leakage and cytosolic Ca^2+^ levels in skeletal muscle (64), indicating that the therapeutic potential of ORAI1 inhibition is not restricted to TAM/STRMK.

ORAI1 is also commonly reported as a target for diseases implicating inflammatory processes, including arthritis, asthma, cancer, conjunctivitis, COVID-19, pancreatitis, pneumonia, psoriasis, or rheumatism (65–67). Several more or less specific ORAI1 inhibitors exist (68–72), and some are currently undergoing clinical trials, as for asthma (RP3128, phase I completed, NCT02958982) (73), COVID-19 pneumonia (Auxora/CM4620, phase II completed, NCT04661540 and NCT04345614) (67, 74), relapsed or refractory lymphomas (RP4010, phase II ongoing, NCT03119467), and acute pancreatitis (Auxora/CM4620, phase II ongoing or completed, NCT03709342, NCT04681066, and NCT03401190). Besides pharmacological ORAI1 inhibitors, alternative strategies employed ORAI1-specific antibodies to reduce autoimmune response in ex vivo T cells from humans and mice (75–78), and the *ORAI1*-specific siRNA SYL116011 is used in preclinical studies for allergic conjunctivitis (79, 80). Altogether, these examples foreground the general physiological importance of SOCE and suggest that efficient ORAI1 inhibitors may be applicable to a vast range of rare and common human diseases.

It is debatable whether compounds specifically targeting ORAI1 or indistinctively all 3 ORAI paralogs represent the most promising strategy since a broader pharmacological range can come along with an increased risk of undesirable side effects. This is probably disorder dependent. Our results on TAM/STRMK mice suggest that the selective inhibition of ORAI1 function is sufficient to antagonize and rescue most disease signs. However, our experiments have been performed on mice with antenatal expression of the ORAI1 R93W mutation and describe the efficiency of ORAI1 inhibition to anticipate disease development. Whether the postnatal administration of ORAI1-specific molecules can effectively attenuate disease progression or revert disease signs remains to be determined.

Considering that ER stress is a major contributor to the muscle phenotype in *Stim1^R304W/+^* mice, any molecule modulating UPR may constitute an alternative therapeutic avenue for TAM/STRMK. As an example, treatment with the chemical chaperone 4-PBA reduced reticular stress and improved skeletal muscle function in mouse models for central core disease and Duchenne muscular dystrophy (81, 82), two disorders involving a similar cellular Ca^2+^ overload as TAM/STRMK.

### Concluding remarks.

The present study provides the proof of concept that the inhibition of the ORAI1 Ca^2+^ channel improves and widely rescues the multisystemic phenotype in *Stim1^R304W/+^* mice, validating ORAI1 as the principal target for the treatment of TAM/STRMK. Small molecules inhibiting SOCE are currently in clinical trials for diverse human diseases, and their verification in *Stim1^R304W/+^* mice might accelerate their accessibility to patients with TAM/STRMK, for which the therapeutic efficacy could be monitored by the circulating biomarker myostatin. Inversely, the discovery of pharmacological compounds antagonizing TAM/STRMK in our *Stim1^R304W/+^* model may also be of medical interest for other Ca^2+^-related disorders.

## Methods

### Sex as biological variable.

With except ion of the bleeding test, all experiments were conducted on male mice. *Stim1^R304W/+^* male mice show an overall stronger and less variable phenotype compared with *Stim1^R304W/+^* female mice.

### Animals.

Mice were housed in ventilated cages with free access to food and water and 12-hour-day/night cycles. *Stim1^R304W/+^* and *Orai1^+/–^* mice (a gift from Paul F. Worley, Johns Hopkins University, Baltimore, Maryland, USA) and *Ora1^R93W/+^* mice (a gift from Stefan Feske, New York University School of Medicine, New York, New York, USA) have been described previously (35, 39, 83). *Stim1^R304W/+^* and *Ora1^R93W/+^* mice (all C57BL/6N) were crossed to generate offspring with the following genotypes: *Stim1^+/+^Ora1^+/+^* (WT), *Stim1^+/+^Ora1^R93W/+^*, *Stim1^R304W/+^Ora1^+/+^*, or *Stim1^R304W/+^Ora1^R93W/+^*. Genotyping primers were GCAGGTAGGAGAGTGTACAGGATGCCTT (forward) and CTTTCCATCCCCACTGCCATTTT (reverse) for *Stim1*, as well as ATTTCCCAATACGTTCCACCTCCC (forward) and TCGTACCACCTTCTTGGGACTTGA (reverse) for *Orai1*. For *Stim1^R304W/+^Ora1^+/–^* mice, the *Orai1* genotyping primers ATGCCTACTGCCAAAATTGAC (forward) and AAATACTGAGCCATCTCTCCTG (reverse) were used. In the main text, *Orai1* downregulation refers to the *Stim1^R304W/+^Ora1^+/–^* group and ORAI1 inhibition to the *Stim1^R304W/+^Ora1^R93W/+^* group.

### Hanging and open-field tests.

To assess general muscle force, mice were suspended upside down on a cage grid for a maximum of 60 seconds, and the latency to fall was recorded. The tests were performed monthly and in triplicate with a 5- to 10-minute rest interval. The open-field test was performed on 10-week-old mice in a homogenously illuminated (100 lux at arena level) and noise-isolated room. The animals were placed in the arena (Bioseb), and rearing, velocity, and covered distance were quantified over 30 minutes.

### In situ muscle force.

To determine maximal and specific muscle force, 4-month-old mice were anesthetized by intraperitoneal injections of a mixture of domitor/fentanyl (2/0.28 mg/Kg), diazepam (8 mg/Kg), and fentanyl (0.28 mg/Kg). The TA was partially excised, and the proximal tendon was attached to an isometric transducer (1305A whole animal system, Aurora Scientific). Maximal force was assessed by sciatic nerve stimulations of 1–200 Hz pulses, spaced by 30 seconds, and fatigue by 80 stimulations of 40 Hz, spaced by 2 seconds. Specific force was determined by dividing the maximal force by the muscle cross sectional area calculated as wet muscle (mg)/optimal muscle length (mm) × mammalian muscle density (1.06 mg/mm^3^). Contraction time corresponds to the time span until maximal muscle force (100%) was reached after single stimulations. Relaxation time reflects the duration of muscle force decrease by 50% after single or tetanic stimulations.

### Micro-computerized bone tomography.

Trabecular bone morphology of the femur from 4-month-old mice was monitored with a Quantum μCT scanner (Perkin Elmer) with an isotropic voxel size of 10 μm, 160 μA tube current, and 90 kV tube voltage. Gray-scale images were preprocessed using ImageJ software (NIH). Morphological 3D measurements and representative images were realized with the CTAn and CTvol software (Bruker), respectively.

### Blood collection, counting, bleeding test, and ELISA assay.

Blood samples were collected in EDTA-coated Microvette 500 K3E tubes (Sarstedt), and blood count was performed on the ADVIA 120 system (Siemens) to determine platelet, erythrocyte, and leukocyte numbers, as well as hemoglobin and hematocrit levels. For the bleeding tests, 4-month-old mice were anesthetized by intraperitoneal injection of ketamine/xylazine (25 km/kg/10 mg/kg). Bleeding was induced by a 3 mm tail-tip transection and monitored until cessation. The test was terminated after 30 minutes if blood flow continued.

Plasma was collected in heparin-coated tubes (Sarstedt), and CK levels were determined using the OLYMPUS AU-480 automated laboratory work station (Beckman Coulter) with adapted kits and controls. The circulating myostatin levels in murine and human plasma samples were assessed with the GDF-8 Quantikine ELISA kit (R&D Systems) according to the manufacturer’s instructions. The myostatin concentration was normalized using the DC Protein Assay kit (Bio-Rad Laboratories).

### Muscle and spleen histology and immunofluorescence.

Muscle specimens from 4-month-old mice were frozen in liquid nitrogen-cooled isopentane, and 8 μm sections were stained with H&E to assess general myofiber morphology and nuclear positioning. The samples were imaged using the Nanozoomer 2HT slide scanner (Hamamatsu), and the individual myofibers were demarcated with the Cellpose segmentation algorithm (84). The MinFeret diameter was calculated with ImageJ, and the number of fibers with nuclear internalization was assessed through the Cell Counter ImageJ plugin.

For immunofluorescence, 8 μm muscle sections were incubated with the following primary and secondary antibodies: mouse anti-human eMHC (F1.652, DHSB), rat anti-mouse CD4 (553727, BD Biosciences), rat anti-mouse CD8 (550281, BD Biosciences), rabbit anti-mouse F4/80 (28463-1-AP, Proteintech), Alexa Fluor 488 goat anti-mouse IgG (115-545-205, Jackson ImmunoResearch), goat anti-rat Alexa Fluor 555 (A21434, Invitrogen), and goat anti-rabbit Alexa Fluor 555 (A32732, Invitrogen). Nuclei were stained with DAPI (Thermo Fisher Scientific) and the sarcolemma with Alexa Fluor 647–conjugated wheat germ agglutinin (W32466, Thermo Fisher Scientific). Images were recorded on an Axioserver microscope (Zeiss), and regenerating fibers were quantified with the ImageJ Cell Counter plugin.

Spleen specimens from 4-month-old mice were fixed in 4% paraformaldehyde and embedded in paraffin, and 5 μm sections were stained with H&E. To determine megakaryocyte numbers, random images covering 12.3 mm^2^ were selected on the Nanozoomer 2HT slide scanner (Hamamatsu) and analyzed with the ImageJ Cell Counter plugin.

### Transcriptomics.

Muscle RNA was extracted from TA at 4 months using TRI Reagent (Molecular Research Center) following the manufacturer’s instructions. For RNA-Seq, the library was generated with the TruSeq Stranded mRNA Sample Preparation Kit, and samples were single-end sequenced on a HiSeq4000 (both from Illumina). Raw data were preprocessed using cutadapt (85), and reads with a Phred quality score above 20 and covering at least 40 nucleotides were mapped onto the mouse genome mm10 assembly using STAR (86). Gene expression was quantified using htseq-count (87) with annotations from Ensembl (http://www.ensembl.org/index.html) and union mode, and normalization and differential gene expression analysis were performed with DESeq2 (88). For the establishment of sample-to-sample distance heatmaps, Euclidean distances were used, and hierarchical clustering was obtained by complete-linkage clustering. Cutoff values for differentially expressed gene determination were as follows: adjusted *P* < 0.05 and absolute value of log_2_FC > 0.5. The rescue status of the genes was determined as described previously (48). Gene ontology analysis was performed with ClusterProfiler (89) by overrepresentation analysis and the Benjamini-Hochberg correction for multiple testing. Enrichments with a corrected *P* < 0.05 were considered significant (89).

For quantitative PCR, RNA was reverse transcribed with the SuperScript IV Transcriptase (Thermo Fisher Scientific), and the cDNA was amplified using the SYBR Green Master Mix I on a LightCycler 480 Real-Time PCR System (both Roche Diagnostics). Forward and reverse primers are listed in Supplemental Table 1. Primer specificity was determined through melting curve analysis and Sanger sequencing of the PCR products. *Rpl27* served as a reference gene (90).

### Ca^2+^ measurements.

Primary myoblasts from 5-day-old mice were isolated as previously described (91), and nonadherent cells were plated on Matrigel Reduced Factor-coated plates (Corning Life Sciences) and cultured in Iscove’s Modified Dulbecco’s Medium (Thermo Fisher Scientific) supplemented with 20% fetal calf serum and 1% chicken embryo extract. Cells were grown and transferred to laminin-coated MatTek dishes and differentiated into myotubes at 70% confluency. Experiments were carried out 4 days after differentiation.

To quantify resting cytosolic Ca^2+^ levels, fully differentiated myotubes were loaded with 5 μM Fura-2/AM (344905, Calbiochem) and washed in Krebs-Ringer solution (120 mM NaCl, 5 mM KCl, 1 mM MgCl_2_, 25 mM NaHCO_3_, and 5.5 mM D-glucose) containing 2 mM Ca^2+^. To induce Ca^2+^ store depletion, the myotubes were incubated in Ca^2+^-free Krebs-Ringer solution supplemented with 0.5 mM EGTA for 5 minutes at 37°C. To induce SOCE, the medium was switched to Krebs-Ringer solution containing 10 mM Ca^2+^, and the changes in cytosolic Ca^2+^ concentrations (Fura-2 fluorescence) were recorded on an Axiovert S100 TV inverted microscope (Zeiss). Data analysis was performed with ImageJ and the Ratio Plus, Process Fura2, and Time Series Analyzer V3 plugins to convert Fura-2 fluorescence to [Ca^2+^] (nM) as previously described (92). The SOCE amplitude reflects the difference in maximal Fura-2 fluorescence ratio (340/380 nm) before and after the switch from Ca^2+^-free to Ca^2+^-containing Krebs-Ringer solution.

### Statistics.

All cell and animal experiments were performed and analyzed in a blinded manner, and the investigators were unaware of the genotype. Normal data distribution was assessed using the Shapiro-Wilk or Kolmogorov-Smirnov test and presented as mean ± SEM. For normally distributed data, we used the 1-way ANOVA followed by Tukey’s post hoc test. Otherwise, the Kruskal-Wallis followed by Dunn’s multiple comparison test was used. The significance of birth ratio was determined by a χ^2^ test, and the significance of myostatin levels was determined by a parametric 2-tailed *t* test. For body weight, hanging time, and force-frequency studies, the 2-way ANOVA followed by Tukey’s post hoc test was used. *P* values of less than 0.05 were considered significant.

### Study approval.

Animal care and experimentation was in accordance with French and European legislation and approved by the Com’Eth ICS-IGBMC institutional ethics committee (Illkirch, France) and validated by the French Ministry of Higher Education and Research and Innovation (project no. 2019062813376603 and 2020052517411298).

Patient blood sampling was performed with written informed consent according to the Declaration of Helsinki and its later amendments. DNA extraction as well as blood/DNA storage and utilization followed institutional IRB-accepted protocols (CE-2022-3) (Medical Faculty, University of Strasbourg). Patients were from France and Spain.

### Data availability statement.

The authors confirm that the data supporting the findings of this study are available within the article and its supplemental material. Data values for all graphs in the manuscript and supplemental material are provided in the Supporting Data Values file. The RNA-Seq data have been deposited in the NCBI’s Gene Expression Omnibus database (GEO GSE244524; https://www.ncbi.nlm.nih.gov/geo/query/acc.cgi?acc=GSE244524).

## Author contributions

RSR, LPG, ST, and AR performed the experiments. RSR, LPG, AS, SD, ST, AR, JL, and JB analyzed the data. AR, LSH, and CT provided biological samples. JL and JB acquired funding and designed and coordinated the study. RSR and JB drafted the manuscript.

## Supplementary Material

Supplemental data

Supporting data values

## Version 1 03/22/2024

Electronic publication
