# Supplementary material for: ORAI1 inhibition as an efficient preclinical therapy for tubular aggregate myopathy and Stormorken syndrome
Source: JCI Insight. 2024 Mar 22;9(6):e174866. doi: 10.1172/jci.insight.174866 (PMC11063934; doi:10.1172/jci.insight.174866)
Supplement: Supplemental data [file jciinsight-9-174866-s272.pdf]

# ORAI1 inhibition as an efficient preclinical therapy for tubular aggregate myopathy and Stormorken syndrome

**Roberto Silva-Rojas, Laura Pérez-Guàrdia, Alix Simon, Sarah Djeddi, Susan Treves, Agnès Ribes, Lorenzo Silva-Hernández, Céline Tard, Jocelyn Laporte, Johann Böhm**

## SUPPLEMENTAL MATERIAL

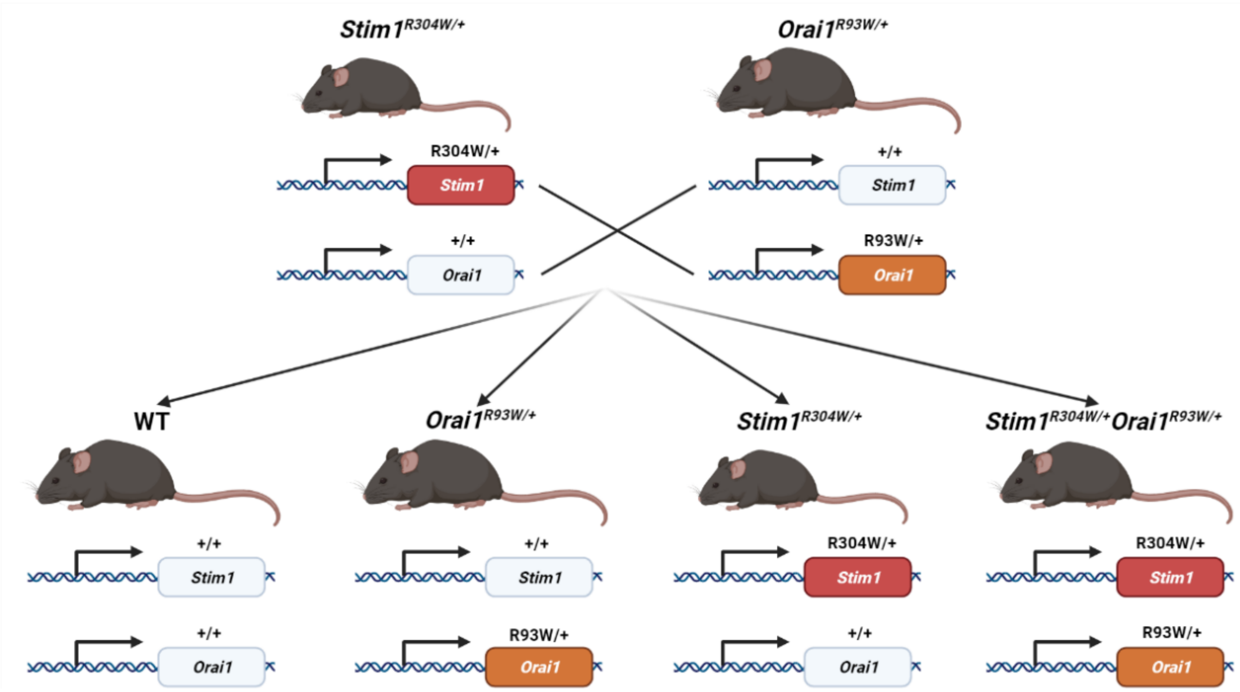


**Figure S1. Crossing scheme.** (A) Crossing of *Stim1*$^{R304W/+}$ and *Orai1*$^{R93W/+}$ mice resulted in offspring with either of the four equiprobable genotypes: WT, *Orai1*$^{R93W/+}$, *Stim1*$^{R304W/+}$, and *Stim1*$^{R304W/+}$*Orai1*$^{R93/W+}$.

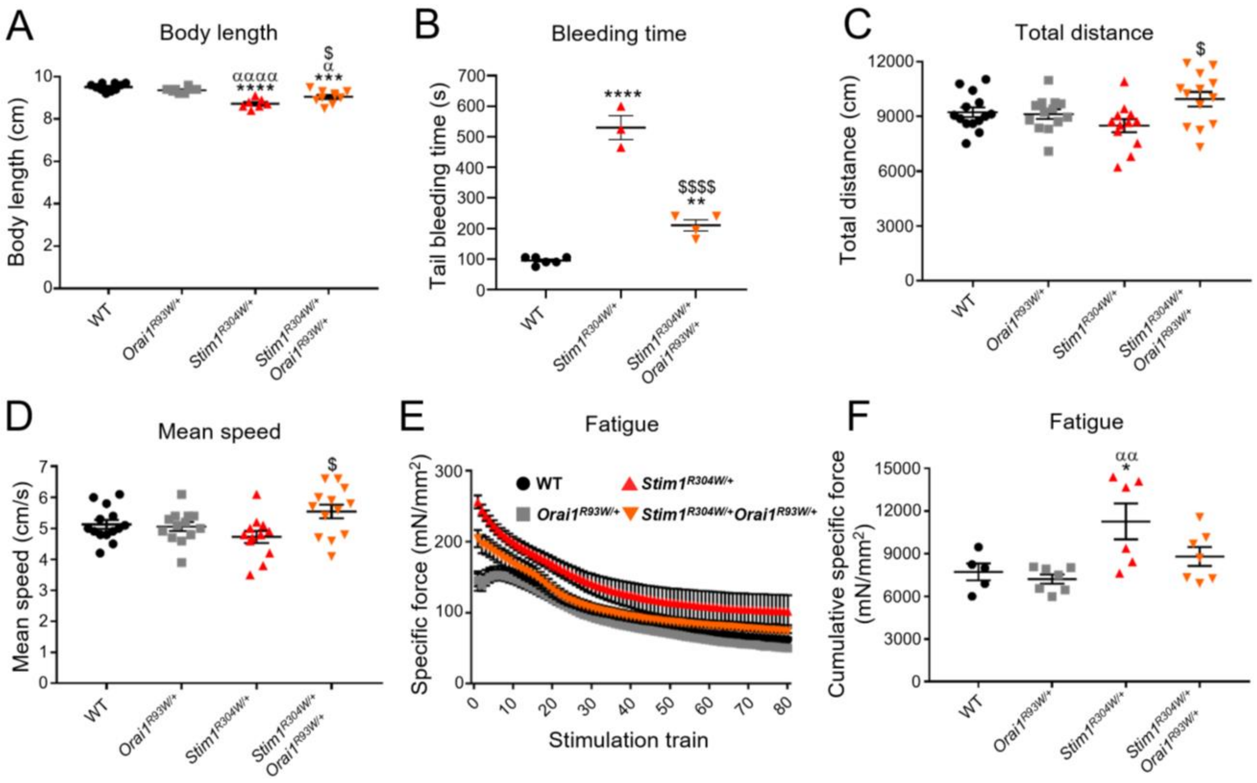


**Figure S2. Improved body length, bleeding times, activity, and fatigue profiles of *Stim1*$^{R304W/+}$*Orai1*$^{R93W/+}$ mice.** (A) At 4 months, *Stim1*$^{R304W/+}$ mice were smaller than WT and *Orai1*$^{R93W/+}$ littermates, and body length was rescued in *Stim1*$^{R304W/+}$*Orai1*$^{R93W/+}$ mice (n=7-10, one-way ANOVA and Tukey's post hoc test). (B) Tail bleeding assays revealed excessive bleeding times in *Stim1*$^{R304W/+}$ mice and significantly improved coagulation in *Stim1*$^{R304W/+}$*Orai1*$^{R93W/+}$ littermates at 4 months (n=3-6, one-way ANOVA and Tukey's post hoc test). (C-D) Open field activity tended to be reduced in *Stim1*$^{R304W/+}$ mice as illustrated by the non-significant decrease of covered distance and mean speed at 10 weeks of age. Both parameters were improved in *Stim1*$^{R304W/+}$*Orai1*$^{R93W/+}$ mice (n=11-14, one-way ANOVA and Tukey's post hoc test). (E-F) At 4 months, *Stim1*$^{R304W/+}$ mice displayed abnormal fatigue curves associated with higher levels of accumulative force compared with WT and *Orai1*$^{R93W/+}$ controls, and both shifted towards normal values in *Stim1*$^{R304W/+}$*Orai1*$^{R93W/+}$ mice (n=5-7, one-way ANOVA and Tukey's post hoc test). Graphs represent mean ± SEM. Significant differences are indicated as */α/$ P<0.05, **/αα/$$ P<0.01, ***/ααα/$$$ P<0.001, and

****/αααα/$$$$ P<0.0001 with * reflecting the comparison of *Stim1*$^{R304W/+}$ with the WT group, α the comparison with the *Orai1*$^{R93W/+}$ group, and $ the comparison with the *Stim1*$^{R304W/+}$*Orai1*$^{R93W/+}$ group.

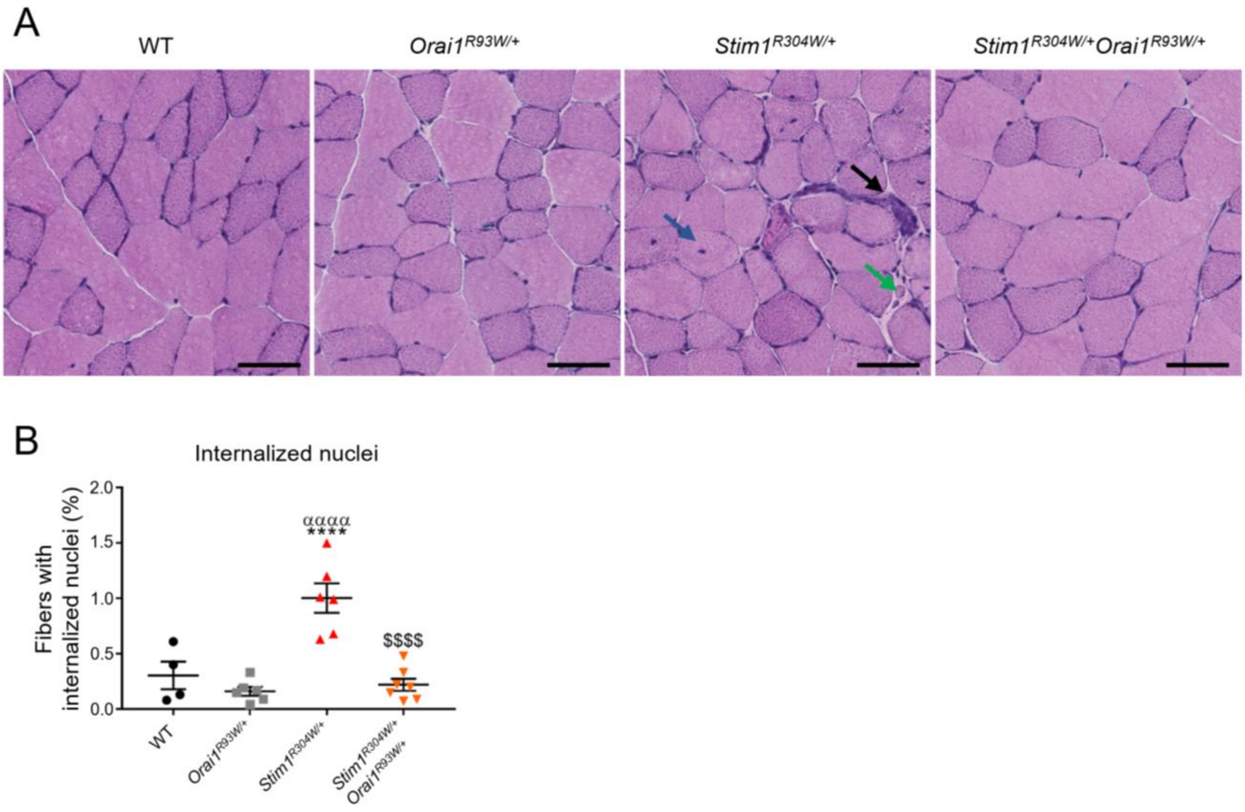


**Figure S3. Resolved myofiber degeneration in *Stim1*$^{R304W/+}$*Orai1*$^{R93W/+}$ gastrocnemius.** (A) Representative H&E pictures of gastrocnemius sections at 4 months showing internalized nuclei (blue arrow), regenerating fibers (green arrow) and infiltration of immune cells (black arrow) in *Stim1*$^{R304W/+}$ mice, and a complete absence of the histopathological hallmarks of muscle fiber degeneration in *Stim1*$^{R304W/+}$*Orai1*$^{R93W/+}$ sections. Scale bar = 50 µm (n=5-7). (B) Fibers with internalized nuclei are increased in *Stim1*$^{R304W/+}$ gastrocnemius and normalized in *Stim1*$^{R304W/+}$*Orai1*$^{R93W/+}$ muscle (n=4-6, one-way ANOVA and Tukey's post hoc test). The graph represents mean ± SEM. Significant differences are indicated as ****/αααα/$$$$ P<0.0001 with * reflecting the comparison of *Stim1*$^{R304W/+}$ with the WT group, α the comparison with the *Orai1*$^{R93W/+}$ group, and $ the comparison with the *Stim1*$^{R304W/+}$*Orai1*$^{R93W/+}$ group.

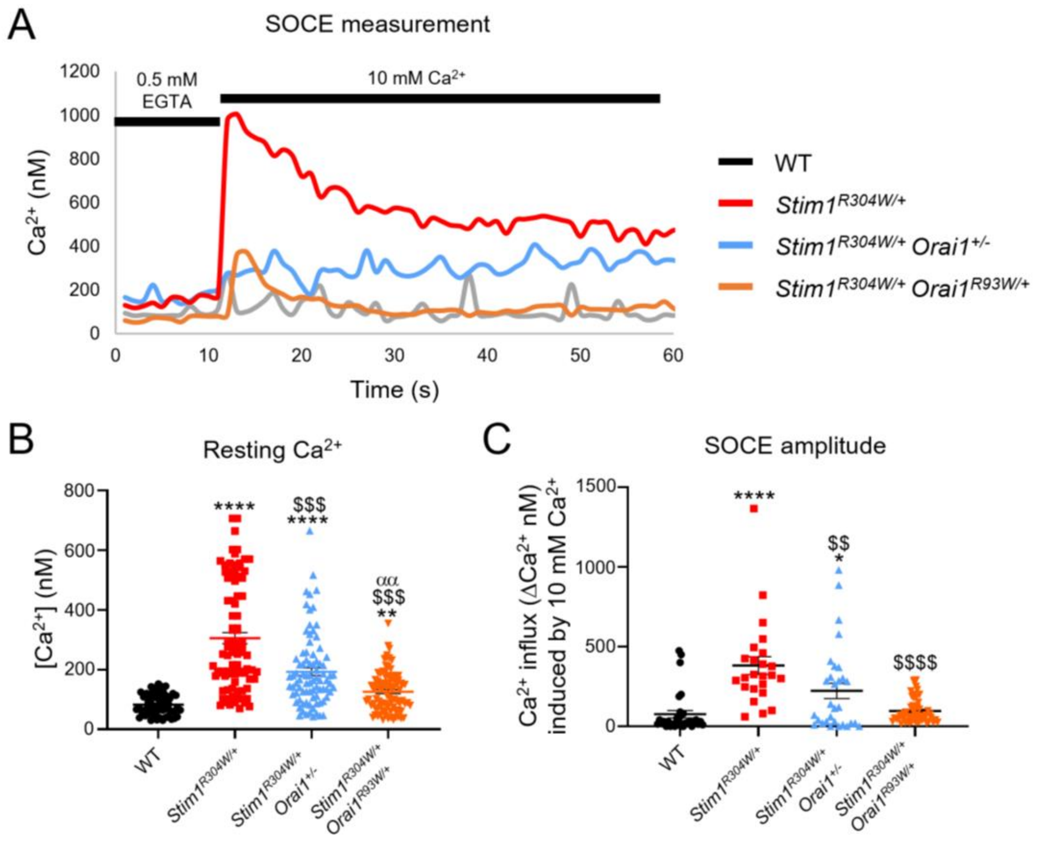


**Figure S4. Comparison of resting $Ca^{2+}$ levels and SOCE amplitude in WT, *$Stim1^{R304W/+}$*, *$Stim1^{R304W/+}Orai1^{+/-}$*, and *$Stim1^{R304W/+}Orai1^{R93W/+}$* myotubes.** (A) Representative traces showing major extracellular $Ca^{2+}$ entry in *$Stim1^{R304W/+}$* myotubes upon addition of 10 mM $Ca^{2+}$ to the medium compared with the WT, and significantly less $Ca^{2+}$ influx in *$Stim1^{R304W/+}Orai1^{+/-}$* and *$Stim1^{R304W/+}Orai1^{R93W/+}$* myotubes (n=24-55 cells). (B-C) Quantification revealed increased resting $Ca^{2+}$ levels and SOCE amplitude in *$Stim1^{R304W/+}$* myotubes compared with the WT. Both parameters were moderately improved in *$Stim1^{R304W/+}Orai1^{+/-}$* myotubes and strongly improved in *$Stim1^{R304W/+}Orai1^{R93W/+}$* myotubes (resting $Ca^{2+}$ n=58-89 and SOCE amplitude n=24-55, Kruskal-Wallis and Dunn's multiple comparison test). Graphs represent mean ± SEM. Significant differences are indicated as **/αα/$$ P<0.01, ***/ααα/$$$ P<0.001, and ****/αααα/$$$$ P<0.0001 with * reflecting the comparison with the WT group, α the comparison with the *$Stim1^{R304W/+}Orai1^{+/-}$* group, and $ the comparison with the *$Stim1^{R304W/+}$* group.

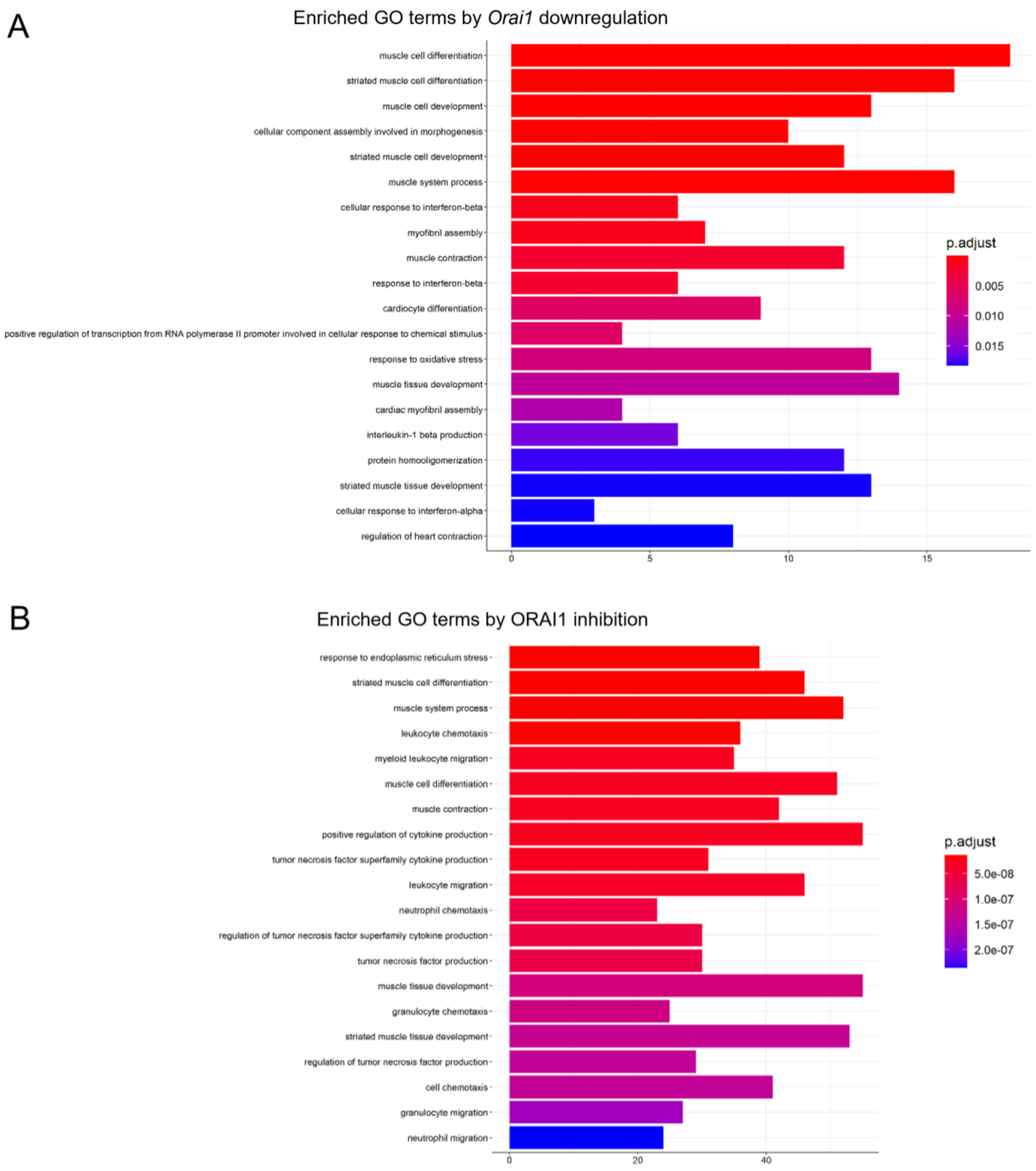


**Figure S5. Comparison of RNA-seq-derived enriched GO terms between ORAI1 inhibition and *Orai1* downregulation.** (A-B) GO term enrichment analysis of genes with partially or completely normalized expression revealed terms associated with muscle contraction and differentiation in both $Stim1^{R304W/+}Orai1^{+/-}$ and $Stim1^{R304W/+}Orai1^{R93W/+}$ mice. Only $Stim1^{R304W/+}Orai1^{R93W/+}$ muscle samples showed an improved expression of genes implicated in immune systems processes and in the response to ER/SR stress (n=4).

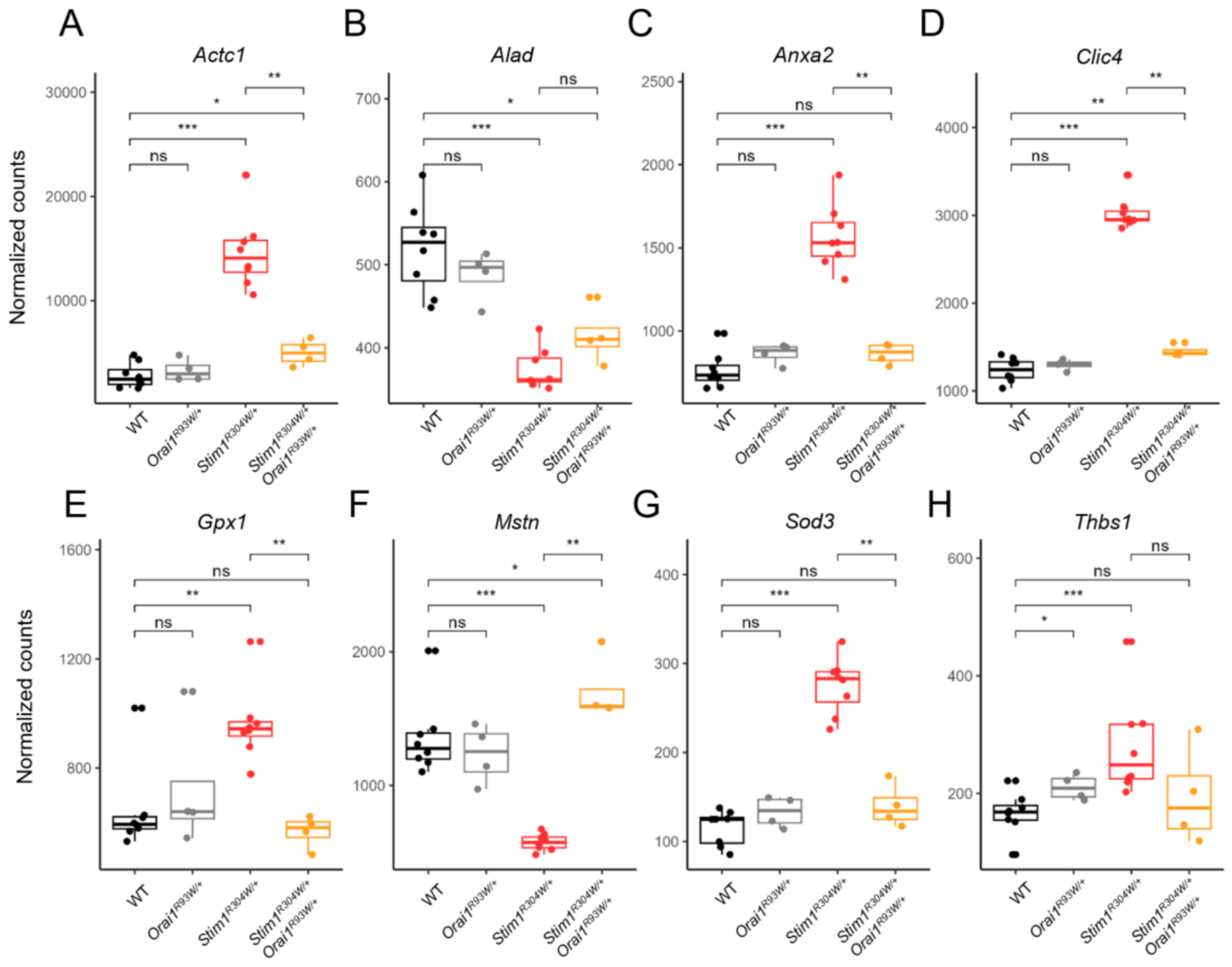


**Figure S6. Selection of *Mstn* as potential biomarker for TAM/STRMK.** (A-H) The known circulating biomarkers *Actc1*, *Alad*, *Anxa2*, *Clic4*, *Gpx1*, *Mstn*, *Sod3*, and *Thbs1* were differentially expressed in *Stim1*$^{R304W/+}$ mice compared with the WT, and all were partially or fully normalized in *Stim1*$^{R304W/+}$*Orai1*$^{R93W/+}$ muscle (n=3-5, one-way ANOVA and Tukey's post hoc test). Graphs represent mean ± SEM. Significant differences are indicated as */α/$ P<0.05, **/αα/$$ P<0.01, and ***/ααα/$$$ P<0.001.

| Gene | Forward primer | Reverse primer |
|---|---|---|
| *Rpl27* | AAGCCGTCATCGTGAAGAACA | CTTGATCTTGGATCGCTTGGC |
| *Hspa5* | CTATTCCTGCGTCGGTGTGT | ATTCCAAGTGCGTCCGATGA |
| *Hspb90b1* | CCACTCAAATCGAACACGGC | AGATTCCGCCTCCTTTCTGC |
| *Spliced Xbp1* | GCTGAGTCCGCAGCAGGT | CAGGCTCCAACTTGTCCAGAAT |
| *Unspliced Xbp1* | CAGACTATGTGCACCTCTGC | CAGGCTCCAACTTGTCCAGAAT |

**Supplemental Table S1. List of RT-qPCR primers**

| | BV/TV (%) | Tb.Th (μm) | Tb.N (1/mm) | Tb.Sp (μm) |
|---|---|---|---|---|
| WT | 5.48 ± 1.03 | 66.08 ± 3.70 | 0.80 ± 0.13 | 455.13 ± 29.63 |
| *Orai1*$^{R93W/+}$ | 3.44 ± 0.44 | 63.39 ± 2.20 | 0.54 ± 0.06 | 538.69 ± 37.32 |
| *Stim1*$^{R304W/+}$ | 0.40 ± 0.10 | 49.66 ± 2.25 | 0.08 ± 0.02 | 770.95 ± 11.96 |
| *Stim1*$^{R304W/+}$*Orai1*$^{R93W/+}$ | 3.55 ± 0.78 | 66.95 ± 2.99 | 0.51 ± 0.10 | 555.80 ± 43.69 |
| p value *Stim1*$^{R304W/+}$*Orai1*$^{R93W/+}$ vs *Stim1*$^{R304W/+}$ | 0.0257 | 0.0012 | 0.0564 | 0.0393 |
| p value WT control vs *Stim1*$^{R304W/+}$*Orai1*$^{R93W/+}$ | >0.999 | 0.9962 | 0.6968 | 0.9156 |
| p value *Orai1*$^{R93W/+}$ control vs *Stim1*$^{R304W/+}$*Orai1*$^{R93W/+}$ | >0.999 | 0.8089 | >0.999 | >0.999 |

**Supplemental Table S2. Trabecular bone parameters of the femur**

BV/TV, bone volume fraction; Tb.Th, trabecular thickness; Tb.N, trabecular number; Tb.Sp, trabecular separation. P values refer to the comparison of *Stim1*$^{R304W/+}$ and *Stim1*$^{R304W/+}$*Orai1*$^{+/-}$ by Tukey's post hoc test one-way ANOVA of all groups (n=6-7).
